# Imaging-guided PCI for event suppression in Japanese acute coronary syndrome patients: community-based observational cohort registry

**DOI:** 10.1007/s12928-020-00649-3

**Published:** 2020-02-12

**Authors:** Takayoshi Yamashita, Kenji Sakamoto, Noriaki Tabata, Masanobu Ishii, Ryota Sato, Suguru Nagamatsu, Kota Motozato, Kenshi Yamanaga, Daisuke Sueta, Satoshi Araki, Yuichiro Arima, Eiichiro Yamamoto, Seiji Takashio, Koichiro Fujisue, Kazuteru Fujimoto, Hideki Shimomura, Ryusuke Tsunoda, Hideki Maruyama, Natsuki Nakamura, Naritsugu Sakaino, Shinichi Nakamura, Nobuyasu Yamamoto, Toshiyuki Matsumura, Ichiro Kajiwara, Shinji Tayama, Tomohiro Sakamoto, Koichi Nakao, Shuichi Oshima, Koichi Kaikita, Seiji Hokimoto, Kenichi Tsujita, Kumamoto Intervention Conference Study (KICS) Investigators

**Affiliations:** 1 Division of Metabolic and Cardiovascular Research, Department of Cardiovascular Medicine, Faculty of Life Sciences, Center for Metabolic Regulation of Healthy Aging, Graduate School of Medical Sciences, Kumamoto University, 1-1-1, Honjo, Chuo-ku, Kumamoto, 860-8556 Japan; 2 Division of Cardiology, National Hospital Organization Kumamoto Medical Center, Kumamoto, Japan; 3 Division of Cardiology, Fukuoka Tokushukai Hospital, Fukuoka, Japan; 4 Division of Cardiology, Kumamoto Red Cross Hospital, Kumamoto, Japan; 5 Division of Cardiology, Minamata City Hospital and Medical Center, Minamata, Japan; 6 Division of Cardiology, Shinbeppu Hospital, Beppu, Japan; 7 Division of Cardiology, Amakusa Regional Medical Center, Amakusa, Japan; 8 Division of Cardiology, Hitoyoshi Medical Center, Hitoyoshi, Japan; 9 Miyazaki Prefectural Nobeoka Hospital, Nobeoka, Japan; 10 Division of Cardiology, Kumamoto Rosai Hospital, Yatsushiro, Japan; 11 Division of Cardiology, Arao City Hospital, Arao, Japan; 12 Division of Cardiology, Kumamoto General Hospital, Yatsushiro, Japan; 13 Cardiovascular Center, Kumamoto Saiseikai Hospital, Kumamoto, Japan; 14 Division of Cardiology, Kumamoto Central Hospital, Kumamoto, Japan

**Keywords:** Acute coronary syndrome (ACS), Intravascular ultrasound (IVUS), Optical coherence tomography (OCT), Percutaneous coronary intervention (PCI), Multi-center registry

## Abstract

Although there is accumulating evidence for the usefulness of imaging-guided percutaneous coronary intervention (PCI), there are few studies for acute coronary syndrome (ACS), and the impact of the frequency of use has not been well addressed. From the Kumamoto Intervention Conference Study; a Japanese registry comprising 17 institutions, consecutive patients undergoing successful PCI from April 2008 through March 2014 were enrolled. Subjects were divided into two groups: imaging-guided PCI and angiography-guided PCI. Clinical outcome was a composite of cardiac death, non-fatal myocardial infarction, and stent thrombosis within 1 year. A total of 6025 ACS patients were enrolled: 3613 and 2412 patients with imaging- and angiography-guided PCI, respectively. Adverse cardiac events were significantly lower in the imaging-guided PCI group (long-rank *P* < 0.001). Even after propensity-score matching, the event rates still showed significant differences between the two groups (log-rank *P* = 0.004). To assess the effects of frequency of imaging usage, we divided the 17 institutions into six low-, six moderate-, and five high-frequency groups. The event rates decreased depending on the frequency, seemingly driven by stepwise event suppression in angiography-guided PCI. In Japanese ACS patients, the incidence of adverse clinical events in patients treated with imaging-guided PCI were significantly lower than that in patients with angiography-guided PCI. Better clinical result was found in the institutions using intravascular imaging more frequently. University Hospital Medical Information Network (UMIN)-CTR (http://www.umin.ac.jp/ctr/). Identifier: KICS (UMIN000015397).

**Electronic supplementary material:**

The online version of this article (10.1007/s12928-020-00649-3) contains supplementary material, which is available to authorized users.

## Introduction

Intravascular imaging during percutaneous coronary intervention (PCI) is a useful tool for providing information on coronary lesion characteristics and the landing site of coronary stents. Intravascular imaging also provides information on postimplantation stent expansion and apposition or possible complications during PCI [1–4]. Intravascular ultrasound (IVUS) or optical coherence tomography (OCT) is mainly used for the guidance of PCI as intravascular imaging modalities.

Although there is accumulating evidence for the usefulness of imaging-guided PCI [5–9], there are few studies evaluating the impact of these modalities during PCI procedures in patients with acute coronary syndrome (ACS) [10, 11]. Therefore, the primary aim of this study was to investigate the usefulness of intravascular imaging during primary PCI for improving clinical outcome in Japanese patients with ACS in a large-scale, multicenter, observational study.

In this multicenter registry, we also analyzed the event rates related to the differences in usage frequency of imaging devices by classifying participating institutions. Thus, the secondary aim of this study was to clarify the relationship between institutional frequency of imaging-guided PCI and clinical outcomes.

## Methods

### Study population

The Kumamoto Intervention Conference Study (KICS) is a multicenter, observational cohort registry enrolling consecutive patients undergoing PCI in 17 centers in Japan (Kumamoto, Miyazaki, Oita, and Fukuoka prefectures). The KICS registry included successful PCI cases including those for native coronary lesion, bypass grafts, in-stent thrombosis or restenosis, and spontaneous coronary dissection, and the exclusion criteria are (1) unsuccessful PCI (unsuccessful coronary stenting, residual 50% or more stenosis of the culprit lesion, and in-hospital death), (2) PCI only with percutaneous old balloon angioplasty (POBA), (3) not on thienopyridines at the time of discharge, and (4) re-intervention case after the first registration. The primary endpoint of the original protocol of KICS was MACE defined as follows: cardiovascular death, non-fatal myocardial infarction, stent thrombosis, revascularization, or stroke during 1-year observational period.

**Fig. 1:**
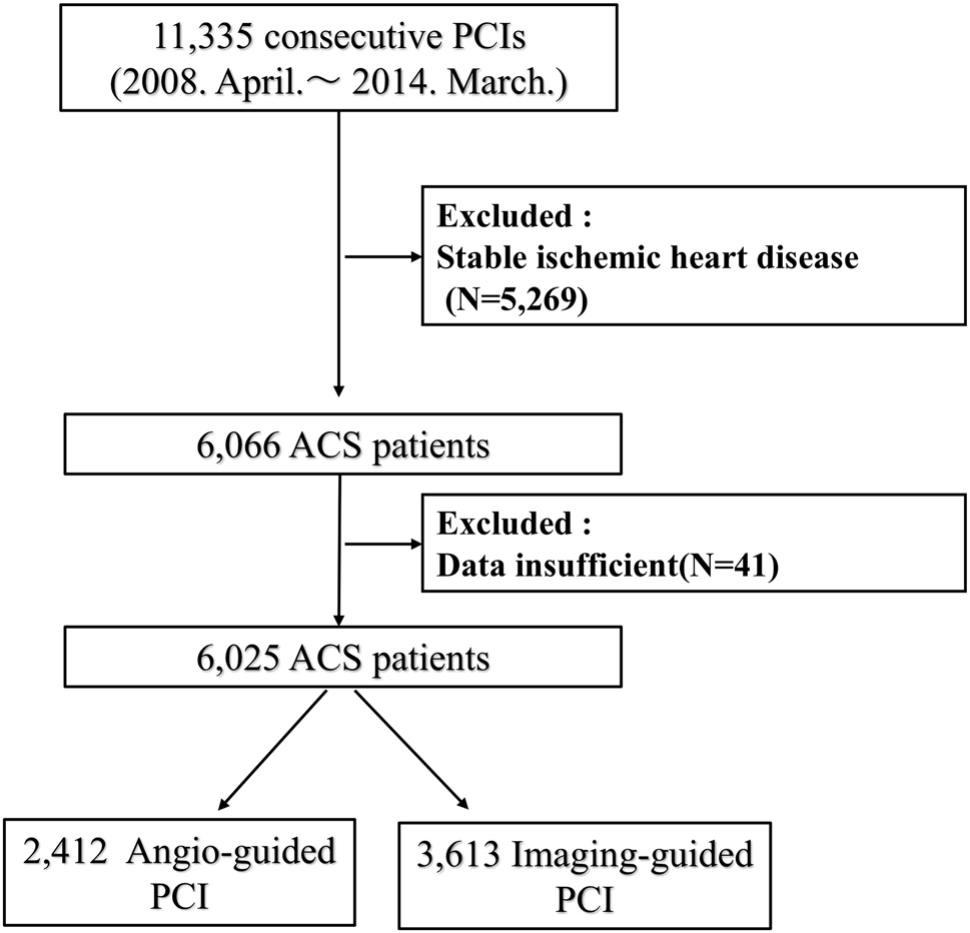
Study flow chart. From 11,335 consecutive patients, we excluded stable ischemic heart disease patients (*N* = 5269) and those with insufficient data (imaging data unknown, *n* = 10; prognosis unknown, *n* = 31). As a result, a total of 6025 ACS patients who met our inclusion criteria were enrolled in this study. *ACS* acute coronary syndrome, *PCI* percutaneous coronary intervention

From April 2008 through March 2014, a total of 11,335 consecutive patients were enrolled in this registry. To elucidate the impact of coronary imaging usage in ACS, we excluded stable ischemic heart disease undergoing PCI and patients with insufficient data such as imaging usage or prognosis data. We divided patients into two groups; the imaging-guided and angiography-guided PCI groups (Fig. 1). In the study protocol, it is allowed to use OCT in addition to IVUS if further evaluation of the culprit lesion is needed. Thus, all patients in the imaging-guided PCI underwent IVUS and some patients are thought to use both IVUS and OCT.

The study complied with the Declaration of Helsinki regarding investigation in humans and was approved by each institutional ethics committee. Informed consent was obtained from all patients.

### Clinical parameters

Baseline demographic data, cardiovascular risk factors, and medications on discharge were documented. ACS was defined as either an acute myocardial infarction (ST-segment elevation myocardial infarction or non-ST-segment elevation myocardial infarction) or unstable angina pectoris. Acute myocardial infarction was diagnosed using the universal definition, in which cardiac troponin is the preferred biomarker of myocardial injury [12], and unstable angina was classified by Braunwald’s classification. We defined diabetes mellitus as a casual plasma glucose concentration ≥ 200 mg/dl, fasting plasma glucose concentration ≥ 126 mg/dl, 2-h plasma glucose concentration ≥ 200 mg/dl from a 75-g oral glucose tolerance test, or taking medication for diabetes mellitus. Hypertension was defined as > 140/90 mmHg or taking antihypertensive medication, and dyslipidemia was defined as low-density lipoprotein ≥ 140 mg/dL (≥ 3.63 mmol/L), high-density lipoprotein < 40 mg/dL (1.04 mmol/L), or triglycerides ≥ 150 mg/dL (≥ 1.7 mmol/L), or taking lipid lowering therapy (i.e., statins). Chronic kidney disease was defined as an estimated glomerular filtration rate < 60 mL/min/1.73 m^2^. Smoking status was determined via interview. Patients with history of endovascular treatment or patients with an ankle-brachial index value of < 0.9 in either leg were categorized as having peripheral arterial disease. Patients with previous ischemic stroke or transient ischemic attack were defined as having cerebrovascular disease. The complexity of lesion was defined by ACC/AHA classification of coronary lesions.

### Primary endpoint in this sub-study during 1-year follow-up

After coronary stent implantation, patients were followed prospectively at outpatient clinics in each institution. Cardiovascular events were ascertained from a review of medical records and confirmed by direct contact with the patients, their families, and physicians. The primary endpoint in the present study was defined as cardiac events comprising cardiac death, non-fatal myocardial infarction, and stent thrombosis within 1 year. Cardiac death included death from MI, stent thrombosis, congestive heart failure, or documented sudden cardiac death. For subjects who had more than one cardiovascular event, only the first event was considered in the analysis.

### Statistical analysis

Continuous variables were expressed as the mean ± SD or median (interquartile range[IQR]). The Shapiro–Wilk test was used to assess the normal distribution of continuous data. Categorical data were presented as numbers or percentages. Differences between two groups were tested using Fisher’s exact test or the Chi-squared test for categorical variables, as appropriate. Differences in continuous variables were analyzed using the analysis of variance or the Kruskal–Wallis test, as appropriate. To estimate the hazard ratios (HRs) and 95% confidence intervals (CIs) for risk factors of cardiovascular event, the Cox frailty model was used with random intercepts to account for institute variation. Multivariable analyses were performed using forced inclusion methods; we selected the variables of conventional risk factors such as age, sex, diabetes, hypertension, dyslipidemia, smoking, and chronic kidney disease (model 1) and variables of statistical significance in the univariable analyses (*P *< 0.05) other than variables that will cause internal correlations (model 2). We used the Kaplan–Meier method to estimate the cardiovascular event probabilities at 365 days and also the log-rank test to compare the distributions of survival times among groups. Landmark analysis was used to determine the time-to-event rates from 0 to 30 days and 30 to 365 days to 1 year. A propensity score was constructed for imaging guidance in a logistic regression model with the following variables: age, sex, diabetes, hypertension, dyslipidemia, smoking, hemodialysis, previous PCI, previous CABG, STEMI, emergent PCI, and bare metal stent. We used Greedy nearest neighbor matching within a caliper width of 0.01 without replacement. In addition, we used the standardized difference to measure the balance of the variables used in calculating the propensity score; standardized mean difference after propensity score matching was less than 0.1 for parameters used for matching. A *P* value < 0.05 was considered to denote statistical significance. The significance level of the pairwise comparison of three groups was adjusted to 0.017 (Bonferroni adjustment). Statistical analyses were performed using IBM SPSS Statistics for Windows, Version 22 (IBM Corp., Armonk, NY, USA).

## Results

### Utility of coronary imaging in overall analysis

From 11,335 consecutive patients, we excluded stable ischemic heart disease patients (*N* = 5269) and those with insufficient data (imaging data unknown, *n* = 10; prognosis unknown, *n* = 31). As a result, a total of 6025 ACS patients who met our inclusion criteria were enrolled in this study: 2412 patients underwent PCI with angiography-guided PCI and 3613 patients with imaging-guided PCI (Fig. 1). Table 1 shows the clinical characteristics for the two groups. Compared with patients treated with angiography-guidance alone, those with imaging-guided PCI were younger, more likely to be smokers, and more frequently presented with hypertension, dyslipidemia, prior PCI, prior CABG, triple vessel disease, left main trunk disease, longer stent length, and larger stent size, and were supported with IABP. They were less likely to be female, to have ST-segment elevation MI, emergent PCI, and been treated with drug-eluting stents.

**Table 1:** Clinical parameters of study participants at baseline stratified by PCI status

	Total (*n* = 6025)	Imaging–guided PCI (*n* = 3613)	Angio–guided PCI (*n* = 2412)	*P*
Age, mean ± SD (year)	69.7 ± 12.2	69.1 ± 12.0	70.6 ± 12.5	< 0.001
Female sex [*n* (%)]	1719 (28.5)	986 (27.3)	733 (30.4)	0.005
Body mass index (kg/m^2^)	23.7 ± 3.5	23.8 ± 3.5	23.6 ± 3.5	0.084
HbA1c (%)	6.4 ± 1.4	6.4 ± 1.4	6.4 ± 1.5	0.61
Diabetes [*n* (%)]	2158 (35.9)	1325 (36.7)	833 (34.6)	0.053
Hypertension [*n* (%)]	4457 (74.0)	2726 (75.4)	1731 (71.8)	0.001
Dyslipidemia [*n* (%)]	3688 (61.2)	2282 (63.2)	1406 (58.3)	< 0.001
Smoking [*n* (%)]	1881 (31.3)	1166 (32.3)	715 (29.7)	0.019
eGFR (ml/min/1.73 m^2^)	65.3 ± 29.6	64.5 ± 31.2	66.1 ± 27.7	0.15
CKD [*n* (%)]	2354 (39.9)	1396 (40.0)	892 (39.0)	0.243
HD, n (%)	181 (3.0)	121 (3.3)	60 (2.5)	0.032
Previous MI [*n* (%)]	777 (12.9)	482 (13.3)	295 (12.2)	0.111
Prior PCI [*n* (%)]	975 (16.2)	629 (17.4)	346 (14.3)	0.001
Prior CABG [*n* (%)]	150 (2.5)	104 (2.9)	46 (1.9)	0.010
Peripheral arterial disease [*n* (%)]	273 (4.5)	165 (4.5)	109 (4.5)	0.513
STEMI [*n* (%)]	3096 (51.4)	1601 (44.3)	1495 (62.0)	< 0.001
Emergent PCI [*n* (%)]	4190 (69.5)	2254 (62.4)	1936 (80.3)	< 0.001
IABP [*n* (%)]	555 (9.2)	360 (10.0)	195 (8.1)	0.007
Triple vessel disease [*n* (%)]	835 (13.9)	568 (15.8)	267 (11.1)	< 0.001
LMT disease [*n* (%)]	347 (5.8)	250 (6.9)	97 (4.0)	< 0.001
Type A	740 (12.5)	447 (12.5)	293 (12.5)	0.488
Type B	4060 (68.4)	2322 (64.7)	1738 (74.2)	< 0.001
Type C	1132 (19.1)	821 (22.9)	311 (13.3)	< 0.001
BMS [*n* (%)]	2880 (47.8)	1340 (37.1)	1540 (63.8)	< 0.001
DES [*n* (%)]	3145 (52.2)	2273 (62.9)	872 (36.2)	< 0.001
Stent length, median [IQR] (mm)	22 [18–28]	23 [18–28]	20 [18–26]	< 0.001
Mean ± SD, mm	23.7 ± 10.5	24.2 ± 10.7	22.9 ± 10.0	< 0.001
Stent size, median [IQR] (mm)	3.0 [2.75–3.5]	3.0 [2.75–3.5]	3.0 [2.75–3.5]	< 0.001
Mean ± SD (mm)	3.1 ± 0.5	3.2 ± 0.5	3.0 ± 0.5	< 0.001

Data are the mean ± standard deviation (SD), median [interquartile range (IQR)], number, or percentage

*eGFR* estimated glomerular filtration rate, *CKD* chronic kidney disease, *HD* hemodialysis, *MI* myocardial infarction, *PCI* percutaneous coronary intervention, *CABG* coronary artery bypass graft, *STEMI* ST-segment elevation myocardial infarction, *IABP* intra-aortic balloon pump, *LMT* left main trunk, *Type A, B, and C* ACC/AHA classification of coronary lesions, *BMS* bare-metal stent, *DES* drug-eluting stent

**Fig. 2:**
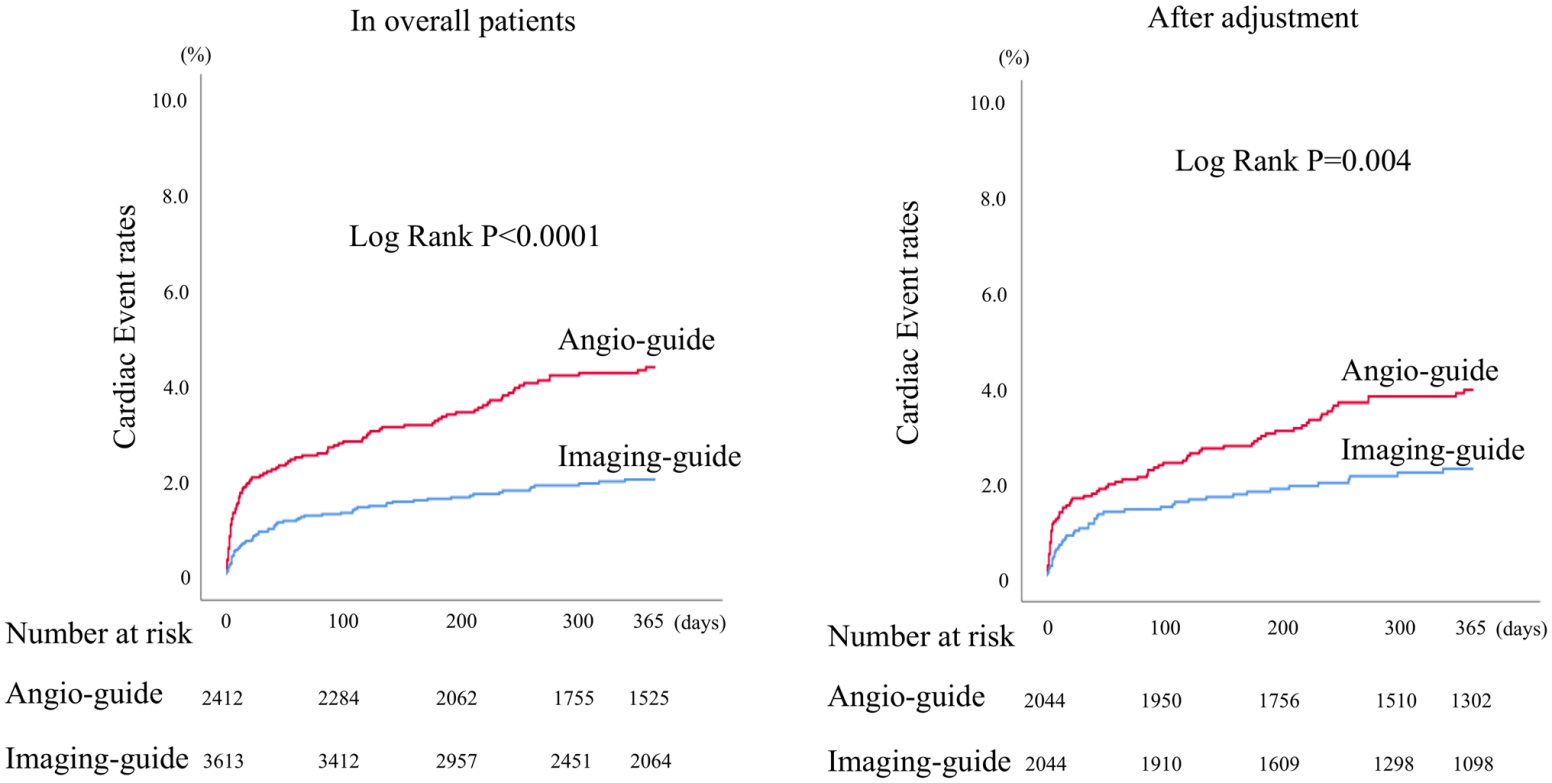
Kaplan-Meier analysis for cardiac events. Patients undergoing imaging-guided PCI had a significantly lower rate of cardiac events than angiography-guided PCI (left). After a propensity matching, cardiac events still showed significant differences between the two groups (right). *PCI* percutaneous coronary intervention

**Fig. 3:**
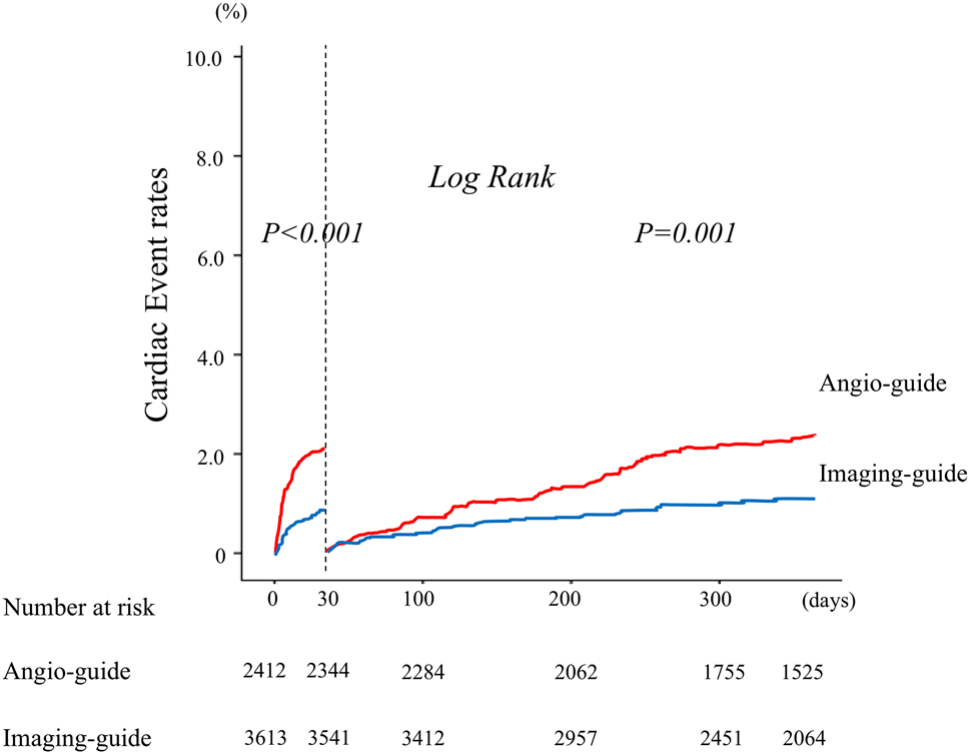
Landmark analysis about cardiac events at 30 days. A Kaplan–Meier analysis for cardiac events in the landmark analysis from discharge to 30 days and from 30 days to 1 year. *PCI* percutaneous coronary intervention

After discharge, a total of 166 (2.8%) patients suffered from a cardiac event within 1-year follow-up period. A Kaplan-Meyer analysis revealed that patients undergoing imaging-guided PCI had a significantly lower rate of cardiac events than angiography-guided PCI (log-rank *P* < 0.001; Fig. 2, left). As detailed in Table 2, the rates of total cardiac events between the imaging-guided and the angiography-guided group were 1.9% and 4.1% (*P* < 0.001), respectively. We observed significantly lower rates of cardiac death (1.3% versus 2.7%; *P* < 0.001), non-fatal MI (0.6% versus 1.5%; *P* < 0.001), and stent thrombosis (0.6% versus 1.2%; *P *= 0.005) in the imaging-guided group than in the angiography-guided group, respectively. The Kaplan–Meier curve for stent thrombosis is shown in Supplemental Figure. We also evaluated the occurrence of cardiac events during two periods, from discharge to 30 days and from 30 days to 1 year, and found that cardiac events were significantly lower in the imaging-guided group during both periods (Fig. 3).

**Table 2:** Cardiac events within 1-year follow-up between imaging-guided and angiography-guided PCI group

	All patents (*n* = 6025)	Imaging-guided PCI (*n* = 3613)	Angio-guided PCI (*n* = 2412)	*P*
Total cardiac events [*n* (%)]	166 (2.8)	67 (1.9)	99 (4.1)	< 0.001
Cardiac death [*n* (%)]	111 (1.8)	47 (1.3)	64 (2.7)	< 0.001
Non-fatal myocardial infarction [*n* (%)]	55 (0.9)	20 (0.6)	35 (1.5)	< 0.001
Stent thrombosis [*n* (%)]	48 (0.8)	21 (0.6)	27 (1.2)	0.017

### Favorable utility of coronary imaging for cardiac events

**Fig. 4:**
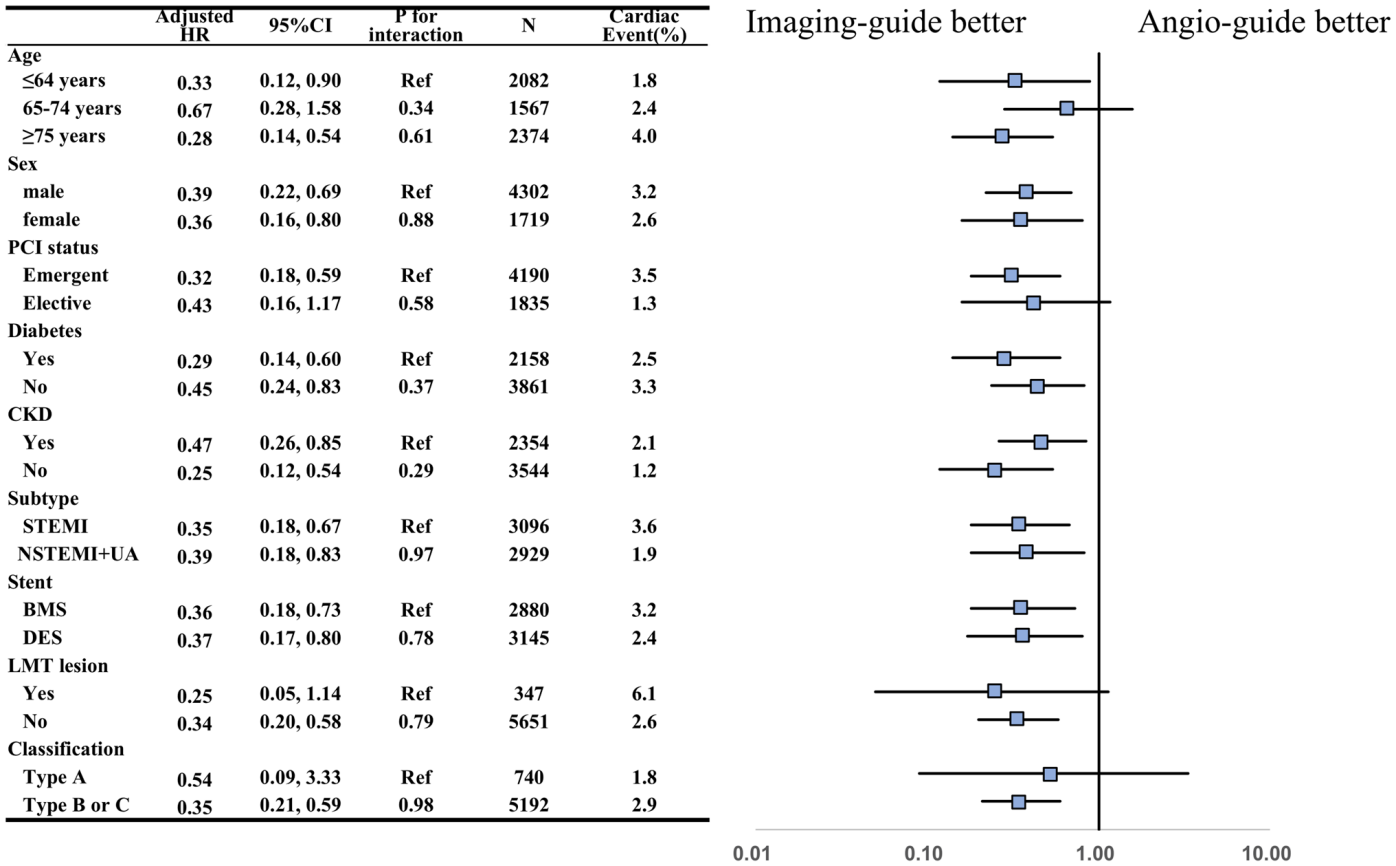
Favorable utility of coronary imaging for cardiac events in relation to patient status. *BMS* bare-metal stent, *CKD* chronic kidney disease, *DES* drug-eluting stent, *LMT* left main trunk, *Types A–C* lesion types defined by American Heart Association classification

In subgroup analysis, we calculated HRs using a multivariable Cox frailty model with random intercepts to account for institute variation. Favorable results for intravascular imaging usage in cardiac events were consistent with both sex, diabetes or not, chronic kidney disease or not, stent type (BMS or DES), and type of MI (STEMI or NSTEMI). In contrast, the advantage of imaging guidance was not observed in patients 65–74 years old (HR 0.67), elective PCI (HR 0.43), left main trunk lesion (HR 0.25), and type A lesion (HR 0.54) (Fig. 4).

### Cox proportional hazard models for predicting cardiac events

Multivariable analyses were performed using forced inclusion methods; we selected the variables of conventional risk factors and imaging usage in model 1. In addition to higher age (HR 1.02, 95% CI 1.00–1.05, *P* = 0.03), imaging-guided PCI was a statistically significant independent negative predictor of cardiac events (HR 0.37, 95% CI 0.24–0.58, *P* = 0.001). In model 2, utilizing variables of statistical significance in the univariable analyses (*P* < 0.05) other than variables that will cause internal correlations, prior MI (HR 1.76, 95% CI 1.12–2.74, *P* = 0.04), STEMI (HR 1.34, 95% CI 1.12–2.74, *P* = 0.014), comorbidity of peripheral artery disease (HR 2.68, 95% CI 1.41–5.09, *P* = 0.003), and imaging guidance (HR 0.41, 95% CI 0.26–0.63, *P* < 0.001) were identified as statistically significant independent predictors of cardiac events (Table 3).

**Table 3:** Cox proportional hazards regression analyses for cardiac events within 1-year follow-up

Variable	Univariable regression			Multivariable Regression			Multivariable Regression		
				Forced Inclusion 1			Forced Inclusion 2		
	HR	95% CI	*P*	HR	95% CI	*P*	HR	95% CI	*P*
Age	1.03	1.02–1.05	< 0.001	1.02	1.00–1.05	0.03	1.01	0.99–1.03	0.17
Male	0.82	0.59–1.13	0.82	1.02	0.63–1.65	0.94			
Diabetes	1.33	0.98–1.81	0.07	1.47	0.96–2.24	0.08			
Hypertension	0.77	0.55–1.06	0.11	0.29	0.49–1.24	0.29			
Hyperlipidemia	0.67	0.49–0.91	0.009	0.83	0.54–1.28	0.40	0.79	0.52–1.21	0.28
Smoking	0.90	0.65–1.26	0.55	1.55	0.94–2.53	0.08			
CKD	1.78	1.78–2.68	0.006	1.56	0.99–2.45	0.053	1.48	0.95–2.31	0.09
HD	1.85	0.95–3.62	0.072						
Emergent PCI	2.84	1.83–4.40	< 0.001						
Prior MI	1.65	1.13–2.42	0.01				1.76	1.12–2.74	0.04
Prior PCI	1.21	0.83–1.78	0.33						
PAD	2.37	1.44–3.92	0.001				2.68	1.41–5.09	0.003
STEMI	1.88	1.36–2.58	< 0.001				1.34	1.12–2.74	0.014
BMS	1.35	0.99–1.82	0.056						
Imaging guided	0.45	0.33–0.61	< 0.001	0.37	0.24–0.58	0.001	0.41	0.26–0.63	<0.001

*CKD* chronic kidney disease, *HD* hemodialysis, *PCI* percutaneous coronary intervention, *MI* myocardial infarction, *PAD* peripheral arterial disease, *STEMI* ST-segment elevation myocardial infarction, *BMS* bare metal stent, *HR* hazard ratio, *CI* confidence interval

### Adjusted outcomes after propensity score matching

A total of 2044 patients treated with imaging guidance were successfully matched to similar patients treated with angiography guidance. We confirmed that the covariate balance in the matched cohort was considerably improved. And in the Hosmer–Lemeshow test, *P* value was 0.983 and the area under the curve of the receiver operating characteristic curve was 0.742. The Hosmer–Lemeshow goodness of fit and discrimination test provided acceptable results. Differences in stent length and size remained after propensity matching; the imaging-guided group was treated by larger and longer stents. In addition, compared with the angiography-guided group, patients in the imaging-guided group were more likely to have three-vessel disease, left main trunk disease. There were also more likely to have been supported by IABP. (Supplemental Table 1)

After propensity score matching, compared with the angiography-guided group, the imaging-guided group was less likely to have cardiac events, non-fatal MI, and stent thrombosis (Table 4). Kaplan–Meier analyses for cardiac events (Fig. 2, right) and stent thrombosis (Supplemental Fig. 1) still showed significant differences between the two groups even after propensity score matching.

**Table 4:** Cardiac events within 1-year follow-up between imaging-guided and angiography-guided PCI groups after propensity matching

	All patents (*n* = 4088)	Imaging-guided PCI (*n* = 2044)	Angio-guided PCI (*n* = 2044)	*P*
Total cardiac events [*n* (%)]	117 (2.9)	42 (2.1)	75 (3.7)	0.001
Cardiac death [*n* (%)]	76 (1.9)	31 (1.5)	45 (2.2)	0.128
Non-fatal myocardial infarction [*n* (%)]	41 (1.0)	11 (0.5)	30 (1.5)	0.005
Stent thrombosis [*n* (%)]	34 (0.9)	11 (0.6)	23 (1.2)	0.037

### Differences among the participating institutions in the utilization rates of coronary imaging and cardiac events

**Fig. 5:**
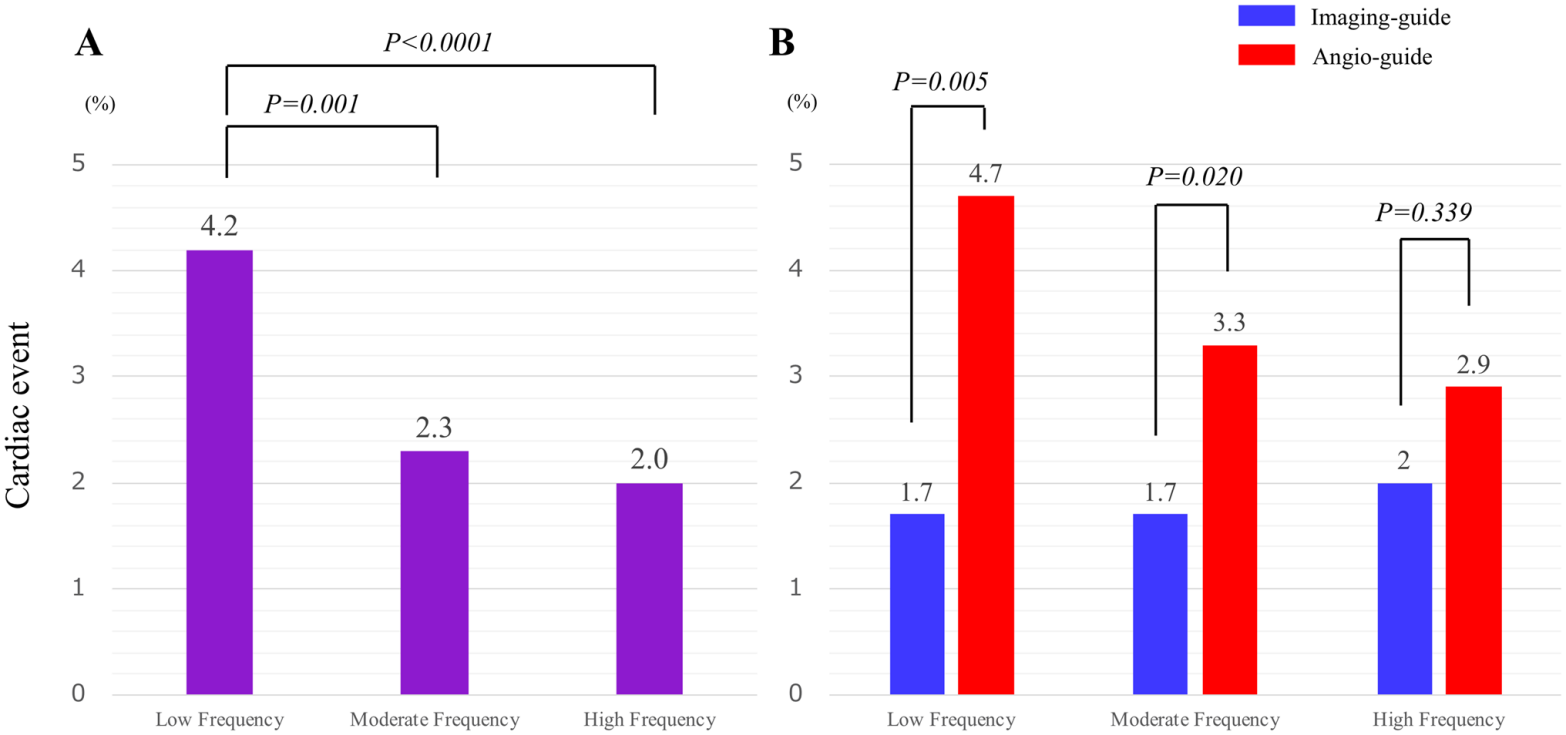
**a** Cardiac event rates among institutions classified by imaging usage frequency. When the institutions were categorized into three groups by the frequency of coronary imaging usage, the incidence of cardiac events decreased stepwise (4.2%, 2.3%, and 2.0%). Adjusted significance level (Bonferroni): 0.017. **b** Cardiac events between imaging-guided and angiography-guided PCI groups among institutions, classified by imaging usage frequency. The event rates of the patients who underwent imaging-guided PCI were comparable. There were statistically significant differences between imaging-guided and angiography-guided PCI groups in the low- and moderate-frequency institutions. Blue bar, imaging-guided PCI; red bar, angiography-guided PCI

In this cohort registry, patients were enrolled from 17 institutions. The utilization rate of intravascular imaging varied widely depending on each institution from 1.4% to 100% (mean 55.7% ± 34.6, median 51.6%) (Supplemental Table 2). When the institutions were categorized into three groups by the frequency of coronary imaging usage (low-frequency institutions: under 33%, six institutions, enrolled 1922 patients; moderate-frequency institutions: 33–90%, six institutions, enrolled 1972 patients; and high-frequency institutions: over 90%, five institutions, enrolled 2131 patients), the incidence of cardiac events decreased stepwise (4.2%, 2.3%, and 2.0%; Fig. 5a). Interestingly, the event rates of the patients who underwent imaging-guided PCI in each group were comparable (1.7%, 1.7%, and 2.0%; Fig. 5b). When comparing cardiac events between imaging guidance and angiography guidance in each categorized group, there were statistically significant differences in the low- and moderate-frequency institutions (*P* = 0.005 and *P* = 0.020, respectively). In contrast, comparable event rates were observed in the high-frequency institutions (*P *= 0.339) (Fig. 5b).

## Discussion

The main findings of the present study were as follows: (1) In Japanese ACS patients, 60% were treated with imaging-guided PCI; (2) adverse cardiac events within a 1-year follow-up were significantly lower in the imaging-guided group than in the angiography-guided group, both in the STEMI and NSTEMI/UAP groups; (3) imaging-guided PCI was an independent and significant predictor of better clinical outcomes, and the favorable impacts of imaging were observed both within the 30 days and during the 30–365 days after PCI, even after propensity score matching; and (4) institutions where coronary imaging is frequently used showed better clinical outcomes than institutions with less frequent use of coronary imaging, seemingly driven by better results in angiography-guided PCI.

In patients with ACS, revascularization with PCI results in improved long-term survival and reduced adverse cardiovascular events [13]. However, despite successful PCI and optimal medical treatment, adverse cardiac events still occur [14]. There is accumulating evidence of the usefulness of coronary imaging in improving clinical outcome [1, 2]. However, the utilization rate of coronary imaging in the clinical setting is not sufficiently high. In a recent report, although not limited to ACS, according to the analysis using the huge Healthcare Cost and Utilization Project’s National Inpatient Sample database in the United States, the usage rate of coronary artery imaging such as IVUS and OCT was only 4.8% of PCIs [15].　Indeed, this retrospective observational study is considered to be an important finding in that coronary imaging was used in 60% of all patients with ACS.

In the Assessment of Dual Antiplatelet Therapy (ADAPT)-DES trial, IVUS guidance was associated with a reduction in stent thrombosis, myocardial infarction, and major cardiac events after DES implantation, and the benefit of IVUS was greater in patients with ACS [16]. In the present study, we also observed significantly lower rates of total cardiovascular events, including cardiovascular death, nonfatal myocardial infarction, and ST-segment elevation in imaging-guided PCI patients.

In the ADAPT-DES study, the prognosis was improved by IVUS guidance between 30 and 365 days, but there was no significant difference within 30 days [16]. Although the results of landmark analysis limited to ACS in the ADAPT-DES study are not available, there is a possibility that prognosis improves within 30 days in this study, obtained by the utilization of coronary imaging because of the procedural advantages for avoiding acute complications or unfavorable findings such as edge dissection, incomplete stent apposition, and high-risk protrusion. Regarding the interval between 30 and 365 days, we could speculate that prognosis could be improved by avoiding late thrombosis because of optimal stent expansion and apposition during the procedure, and/or by aggressive risk reduction therapy through imaging-derived plaque assessment. Considering these factors, it seems that using coronary imaging for intervention in patients with ACS can contribute to improving both short- and long-term prognosis.

Even after propensity score matching (*n* = 2044 in each group), complete patient background matching was inherently impossible in this retrospective observational registration study. In the matched imaging-guided PCI cohort, longer stent, greater use of IABP, more than three-vessel disease, and more left main trunk lesions were observed. The demonstration of the utility of the imaging-guided PCI in this more complicated situation resulted in more emphasis on the effectiveness of coronary imaging.

There are several possible advantages in imaging-guided PCI, though we did not analyze the morphological or histopathological mechanism. It has been reported that early stent thrombosis after primary PCI in patients with ACS was associated with small luminal areas at the stent edge or within the stent, derived from under-expansion of the stent [17, 18]. In this study, average stent size was larger in the imaging-guided group, even after propensity score matching. The favorable results of the imaging-guided group might be derived from the greater lumen gain, resulting from the accurate measurements of coronary lumen as well as vessel size by coronary imaging. In addition, coronary imaging may be useful to identify the appropriate lesion length, resulting in accurate decision of the landing zone before stenting. This may result in the longer stent selection in the imaging-guided group.

In this research, we also focused on the difference in the incidence of cardiac events among various institutions with different usage frequencies of coronary imaging. As far as we know, this is the first report to address the differential event rates according to the imaging-guided PCI frequencies. As shown in the Fig. 5a, stepwise event suppressions were observed when institutions were divided according to the frequency of the imaging-guided PCI. Moreover, the impact of imaging was greater in low- and moderate-frequency institutions rather than higher frequency institutions (Fig. 5b). These results might suggest that frequent imaging users lead to more favorable event suppressions following PCI and that the clinical benefit of coronary imaging with regard to the event suppression could be obtained even in the lower frequency institutions. There is a possibility that frequent imaging users might apply their usual coronary imaging experiences to the angiography-guided PCI with imaging-angiography interpretation feedback, when undertaking PCI without imaging. However, the present study is not a randomized one and it is not possible to confirm the causal effect of frequency imaging usage on institutional event rates. Further prospective studies are expected to confirm our findings.

This study has some limitations. First, this study was a non-randomized observational study. Significant differences in the background characteristics may not justify direct comparisons and the existence of inherent selection bias cannot be denied. Second, it included only Japanese patients. Thus, our results might not be applicable to different ethnic populations all over the world. Third, the present study is a multicenter study, and detailed information of coronary imaging findings such as culprit and non-culprit plaques is not included in the results. Furthermore, the detailed number of the OCT usage is unclear and its impact on clinical outcome is not evaluated in the present study. Moreover, the follow-up was performed at each institution and the information for some visits interval is lacking. In this study, 3759 patients (62.4%) completed 1-year follow-up. There was possibility that the low follow-up rate of the registered patients may have caused some bias. We have additionally performed survival analyses at 180 days’ follow-up (follow up rate was 91.0%). The consistent findings compared with 1-year analysis were observed in Kaplan-Meyer analysis (Supplemental Fig. 2, left) and in multivariable analyses (Supplemental Fig. 2, right).

## Conclusion

In Japanese ACS patients treated with imaging-guided PCI, the rates of adverse cardiovascular events were significantly lower than with angiography-guided PCI.

Imaging-guided PCI was effective regardless of the frequency of its use. Furthermore, imaging-guided PCI frequency might be associated with better clinical outcome also in patients undergoing angiography-guided PCI.

## Electronic supplementary material

Below is the link to the electronic supplementary material.

Supplementary material 1 (DOCX 31 kb)

Supplement Figure 1. Kaplan–Meier analysis for stent thrombosis. Kaplan–Meier curves for stent thrombosis between angiography-guided and imaging guided PCI groups are shown in over all patients (left) and matched cohorts (right)

Supplement Figure 2. Kaplan–Meier analysis for cardiac events at 180 days follow-up and multivariable analyses
