# Supplementary material for: Imaging-guided PCI for event suppression in Japanese acute coronary syndrome patients: community-based observational cohort registry
Source: Cardiovasc Interv Ther. 2020 Feb 12;36(1):81–90. doi: 10.1007/s12928-020-00649-3 (PMC7829241; doi:10.1007/s12928-020-00649-3)
Supplement: Supplementary file 1 — Supplementary material 1 (DOCX 31 kb) [file 12928_2020_649_MOESM1_ESM.docx]

| Supplemental Table 1. Patient characteristics after propensity matching. | | | |
| --- | --- | --- | --- |
|  | Imaging-guided PCI  (n = 2044) | Angio-guided PCI  (n = 2044) | P |
| Age, mean±SD, year | 69.6±12.3 | 69.5±12.4 | 0.732 |
| Female sex, n (%) | 578 (28.3) | 576 (28.2) | 0.478 |
| Body mass index, kg/m^2^ | 23.8±3.5 | 23.8±3.5 | 0.096 |
| Diabetes, n (%) | 727 (35.6) | 732 (35.8) | 0.443 |
| Hypertension, n (%) | 1476 (72.2) | 1479 (72.4) | 0.472 |
| Dyslipidemia, n (%) | 1216 (59.5) | 1229 (60.1) | 0.351 |
| Smoking, n (%) | 628 (30.7) | 637 (31.2) | 0.393 |
| CKD, n (%) | 804 (40.9) | 745 (38.3) | 0.049 |
| HD, n (%) | 58 (2.8) | 56 (2.7) | 0.462 |
| Previous MI, n (%) | 247 (12.1) | 263 (12.9) | 0.239 |
| Previous PCI, n (%) | 318 (15.6) | 313 (15.3) | 0.431 |
| Previous CABG, n (%) | 39 (1.9) | 44 (2.2) | 0.329 |
| Peripheral arterial disease, n (%) | 75 (3.7) | 94 (4.6) | 0.078 |
| STEMI, n (%) | 1170 (57.2) | 1161 (56.8) | 0.400 |
| Emergent PCI, n (%) | 1554 (76.0) | 1574 (77.0) | 0.242 |
| IABP, n (%) | 241 (11.8) | 161 (7.9) | <0.001 |
| 3Vessel disease, n (%) | 299 (14.7) | 243 (12.0) | 0.006 |
| LMT disease, n (%) | 134 (6.6) | 87 (4.3) | 0.001 |
| BMS, n (%) | 1179 (57.7) | 1178 (57.6) | 0.500 |
| DES, n (%) | 865 (42.3) | 866 (42.4) | 0.500 |
| Stent length, median [IQR], mm | 22 [16-28] | 20 [18-26] | 0.093 |
| mean±SD, mm | 23.5±9.9 | 23.1±10.3 | 0.262 |
| Stent size, median [IQR], mm | 3.0 [3.0-3.5] | 3.0 [2.75-3.5] | <0.001 |
| mean±SD, mm | 3.2±0.5 | 3.0±0.5 | <0.001 |

Abbreviations as in Table 1

| **Supplemental Table 2.**  **Imaging usage rate and number of enrolled patients by institution**. | | |
| --- | --- | --- |
| Institution | N | Imaging usage (%) |
| 1 | 140 | 1.4 |
| 2 | 269 | 5.2 |
| 3 | 584 | 14.2 |
| 4 | 330 | 18.8 |
| 5 | 92 | 26.1 |
| 6 | 507 | 32.1 |
| 7 | 113 | 36.3 |
| 8 | 112 | 36.6 |
| 9 | 128 | 51.6 |
| 10 | 1494 | 66.1 |
| 11 | 29 | 75.9 |
| 12 | 96 | 82.3 |
| 13 | 1096 | 94 |
| 14 | 171 | 94.7 |
| 15 | 634 | 96.4 |
| 16 | 193 | 97.9 |
| 17 | 37 | 100 |
