# Supplementary figures and images for: Imaging-guided PCI for event suppression in Japanese acute coronary syndrome patients: community-based observational cohort registry
Source: Cardiovasc Interv Ther. 2020 Feb 12;36(1):81–90. doi: 10.1007/s12928-020-00649-3 (PMC7829241; doi:10.1007/s12928-020-00649-3)

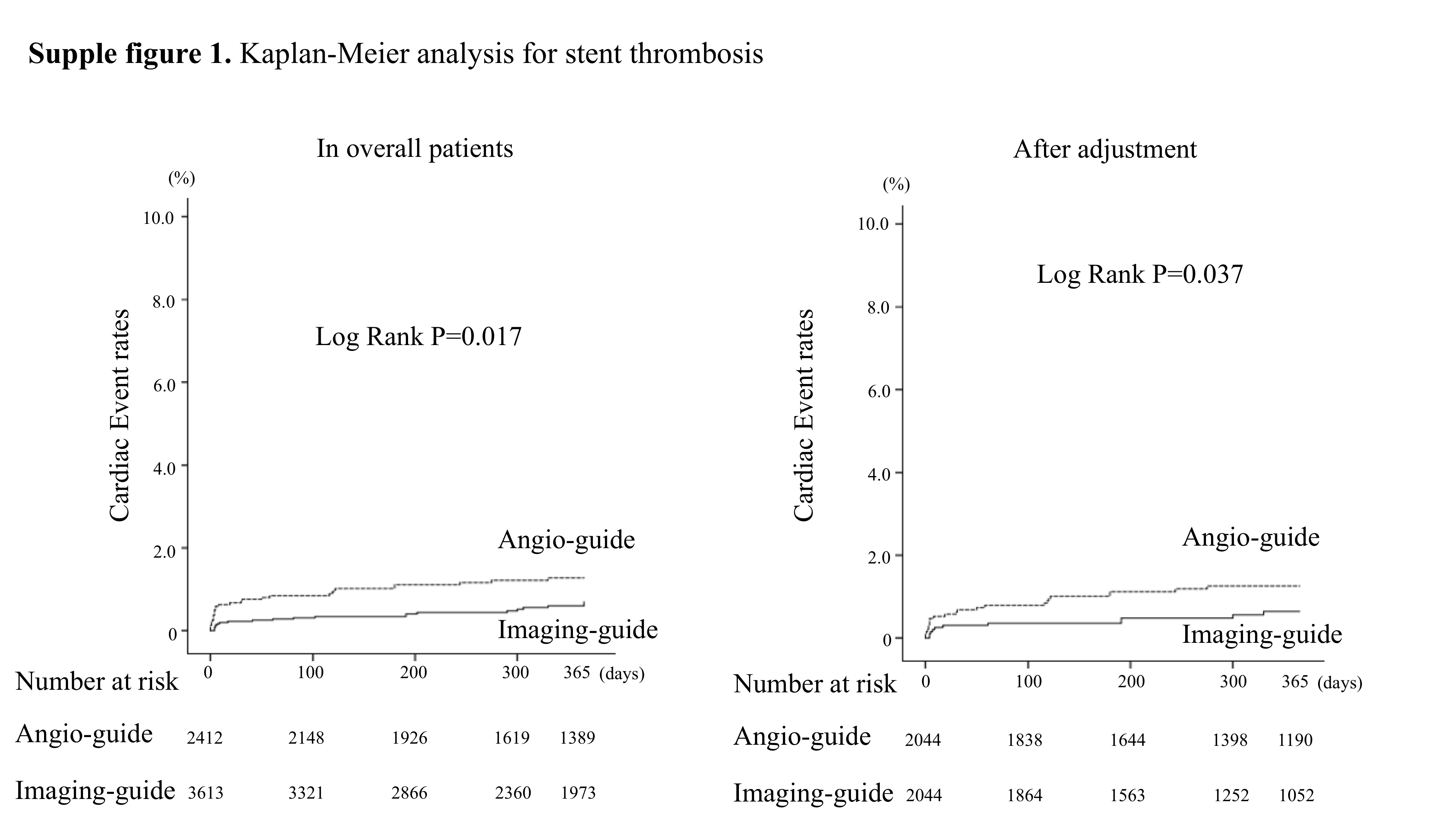

Supplement: Supplementary file 2 — Supplement Figure 1. Kaplan–Meier analysis for stent thrombosis. Kaplan–Meier curves for stent thrombosis between angiography-guided and imaging guided PCI groups are shown in over all patients (left) and matched cohorts (right) [file 12928_2020_649_MOESM2_ESM.tiff]

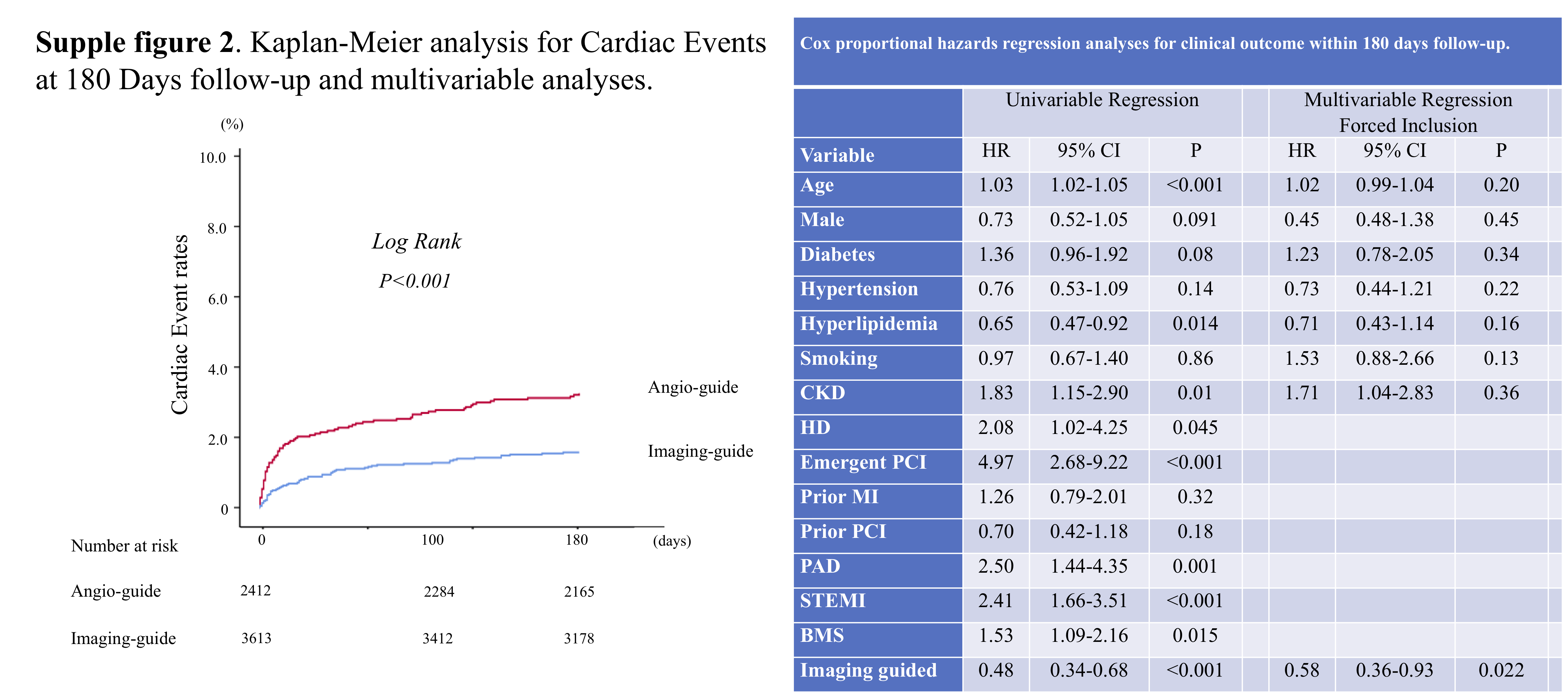

Supplement: Supplementary file 3 — Supplement Figure 2. Kaplan–Meier analysis for cardiac events at 180 days follow-up and multivariable analyses [file 12928_2020_649_MOESM3_ESM.tiff]
